# Combined Treatment with Three Natural Antioxidants Enhances Neuroprotection in a SH-SY5Y 3D Culture Model

**DOI:** 10.3390/antiox8100420

**Published:** 2019-09-20

**Authors:** Pasquale Marrazzo, Cristina Angeloni, Silvana Hrelia

**Affiliations:** 1 Department for Life Quality Studies, Alma Mater Studiorum, University of Bologna, 47921 Rimini, Italy; pasquale.marrazzo2@unibo.it; 2 School of Pharmacy, University of Camerino, 62032 Camerino, Italy; silvana.hrelia@unibo.it

**Keywords:** neurodegeneration, SH-SY5Y cell line, 3D cultures, oxidative stress, phytochemicals, antioxidants

## Abstract

Currently, the majority of cell-based studies on neurodegeneration are carried out on two-dimensional cultured cells that do not represent the cells residing in the complex microenvironment of the brain. Recent evidence has suggested that three-dimensional (3D) *in vitro* microenvironments may better model key features of brain tissues in order to study molecular mechanisms at the base of neurodegeneration. So far, no drugs have been discovered to prevent or halt the progression of neurodegenerative disorders. New therapeutic interventions can come from phytochemicals that have a broad spectrum of biological activities. On this basis, we evaluated the neuroprotective effect of three phytochemicals (sulforaphane, epigallocatechin gallate, and plumbagin) alone or in combination, focusing on their ability to counteract oxidative stress. The combined treatment was found to be more effective than the single treatments. In particular, the combined treatment increased cell viability and reduced glutathione (GSH) levels, upregulated antioxidant enzymes and insulin-degrading enzymes, and downregulated nicotinamide adenine dinucleotide phosphate (NADPH) oxidase 1 and 2 in respect to peroxide-treated cells. Our data suggest that a combination of different phytochemicals could be more effective than a single compound in counteracting neurodegeneration, probably thanks to a pleiotropic mechanism of action.

## 1. Introduction

**Figure 1:**
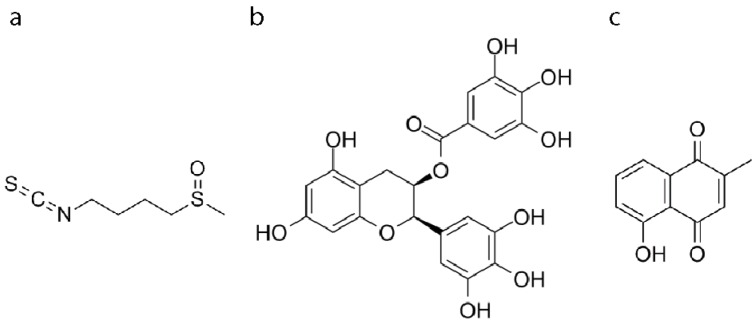
Chemical structures of the natural compounds used in this study. (**a**) Sulforaphane (SF), (**b**) epigallocatechin gallate (EGCG), (**c**) plumbagin (PB).

Oxidative stress is strongly involved in the pathogenesis of different neurodegenerative diseases like Alzheimer’s disease, Parkinson’s disease, and amyotrophic lateral sclerosis [1]. Particularly, an excess of reactive oxygen species (ROS) released by cells promotes oxidative stress, which is a cause of tissue injury and results in dysfunction in the nervous system. So far, no drugs have been discovered to prevent or halt the progression of these widely spread neurological disorders, and the treatments available only manage the symptoms. Therefore, there is an urgent need for new treatments for these diseases, since the World Health Organization (WHO) predicts that by 2040, neurodegenerative diseases will become the second-most prevalent cause of death [2]. New therapeutic approaches can derive from phytochemicals, a huge source of compounds that have been widely investigated in the last years [3]. Sulforaphane (SF) (Figure 1a) is an isothiocyanate derived from *Brassicae* vegetables and has consistent support in the literature for its preventive role against oxidative stress [4], in addition to its well-known role in chemoprevention [5]. Epigallocatechin gallate (EGCG) (Figure 1b), the major catechin found in green tea [6] with diverse medical potential [7] has been demonstrated to promote neuroprotection in numerous studies [8]. Plumbagin (PB) (Figure 1c), a naphthoquinone isolated from the *Plumbaginacae* family, has mainly been studied in respect to its anti-inflammatory [9,10,11] and antimicrobial activities [12], even though it was also shown to modulate oxidative stress response [13] and precisely target NADPH oxidase 4 (NOX4) [14]. Importantly, the treatment of undifferentiated SH-SY5Y cells with a specific concentration of SF [15], EGCG [16], and PB [17] has been reported to decrease oxidative stress at different levels. On this basis, we hypothesized that a proper combination of such bioactive compounds could possess a higher effect in counteracting oxidative stress-induced neurodegeneration. Within organs, there are various concentration gradients for oxygen as well as for effector molecules, i.e., internal metabolites or exogenous compounds/drugs. Since the human brain cannot be modelled adequately in animals [18], reductionist humanized cellular systems are used and increasingly requested according to the 3Rs (Replacement, Reduction and Refinement) rule. 

Currently, the majority of cell-based studies on neurodegeneration have been carried out on cultured cells propagated in two dimensions on plastic surfaces. However, cells cultured in these non-physiological conditions do not represent the cells residing in the complex microenvironment of the brain. With respect to this, recent evidence has suggested that three-dimensional (3D) in vitro microenvironments may better model key features of brain tissues in order to study the molecular mechanisms at the base of neurodegeneration and neurorepair [19,20]. Numerous in vitro approaches have been carried out to mimic human neuronal features, based on neuronal-like cells such as the neuroblastoma line SH-SY5Y. SH-SY5Y is a human cell line that divides quickly and has the ability to differentiate in post-mitotic neurons, thus it is considered a convenient model to study Parkinson’s [21] and Alzheimer’s diseases [22]. Unlike traditional two-dimensional (2D) cultures, the different availability of oxygen and growth factors in a 3D cell culture should expectantly favor a more in vivo-like morphology and growth of these cells. Indeed, several 3D culture models have been developed with SH-SY5Y cells, in terms of cell aggregates [23], spheroids [24,25], or including different scaffolds [26,27,28]. To support 3D cultures of SH-SY5Y or neuronal cell lines, collagen has also been used, like collagen hydrogel [29] or porous collagen-based scaffolds [26,30]. To the best of our knowledge, none of the previous models has been used to investigate the ability of natural bioactive molecules to confer resistance to oxidative stress. The aim of this study was to evaluate the neuroprotective effect of a combination of SF, EGCG, and PB in preventing cell damage derived from oxidative stress in a 3D cell culture based on a collagen porous scaffold. 

## 2. Materials and Methods

### 2.1. Cell Culture and Treatment

The SH-SY5Y human neuroblastoma cell line was obtained from Sigma-Aldrich (cat. n° 94030304) (St. Louis, MO, USA). Cells were expanded in a growth culture medium composed of high glucose Dulbecco’s Modified Eagle’s medium (DMEM), supplemented with 10% fetal bovine serum (FBS), 2 mM glutamine, 50 U/mL of penicillin, and 50 μg/mL of streptomycin, and cultured at 37 °C with 5% CO_2_ as previously reported [31]. Cell differentiation was induced by reducing serum levels of the medium to 1% with 10 µM retinoic acid (RA) for seven days prior to treatments [32]. SF (LKT Laboratories, Minneapolis, MN, USA), EGCG and PB (Sigma-Aldrich, St. Louis, MO, USA) were dissolved in DMSO, and 10 mM stocks were kept at −20 °C until use. Differentiated SH-SY5Y cells were treated with 1 μM SF, 2.5 μM EGCG, and 0.5 μM PB and SEP (1 μM SF + 2.5 μM EGCG + 0.5 μM PB,) for 24 h or 6 h according to the different experiments. Oxidative stress was induced, as previously reported [33], by exposing cells to 700 µM H_2_O_2_ in 1% FBS DMEM. 

### 2.2. 3D Model Preparation

To obtain the scaffolds for 3D cultures of SH-SY5Y cells, sterile heterologous native lyophilized collagen type I sponge (BIOPAD™, Angelini Pharma Inc., Gaithersburg, USA) was cut using a sterile scalpel into pieces with squared dimensions able to fit 96-multiwell culture plates. Each piece was divided by subjecting it to a second longitudinal cut, performed in order to present a similar top surface as the cells. The pieces with approximately 1 cm^2^ of surface area were inserted into a 24-multiwell plate and constituted the scaffolds for the cell culture. To establish the 3D SH-SY5Y culture, 50 μL of cell suspension in DMEM with 10% FBS was seeded atop of each scaffold. Different cell numbers per scaffold (50 × 10^3^–100 × 10^3^–200 × 10^3^) were seeded to compare cell viability along the culture (1–6 days) and optimize cell seeding for differentiation. To differentiate 3D SH-SY5Y culture, a concentration of 4 × 10^6^ cells/mL equivalent to 200 × 10^3^ cells in 50 uL was used. After 45 min of incubation at 37 °C, 5% CO_2_, DMEM with 1% FBS, and 10 µM RA were added to the 3D culture in order to induce cell differentiation. The medium was changed every two days. 

### 2.3. MTT Assay

Before adding 3-(4,5-Dimethylthiazol-2-yl)-2,5-diphenyl-tetrazolium bromide (MTT), 3D cultures were transferred to clean cell culture wells. MTT 0.5 mg/mL was prepared in a cell medium and added to the 3D cultures. To measure the percentage of cells that did not attach to the scaffold, MTT was also added to the wells where cells were initially seeded (Figure S1). The MTT solution was incubated for 2 h at 37 °C, 5% CO_2_. After removing the MTT solution, DMSO was added and the absorbance of formazan was measured at 595 nm using a microplate spectrophotometer VICTOR3 V Multilabel plate-reader (PerkinElmer, Wellesley USA). The sum of the two respective absorbance values, the first deriving from the primary wells used during the seeding and the second deriving from the scaffolds, were considered as 100%. 

### 2.4. Prestoblue Assay

A Prestoblue^®^ working solution was prepared in a growth culture medium without phenol red according to the manufacturer’s instructions. Briefly, the culture medium was removed from cell culture wells and a Prestoblue working solution was added and incubated at 37 °C, 5% CO_2_. After 3 h, the well volumes were collected in a new 96-well plate and the absorbance was read at λ = 570 nm (experimental) and λ = 600 nm (reference wavelength for normalization) using a Victor Multilabel plate-reader (Perkin-Elmer, Wellesley USA). 

### 2.5. Reduced Glutathione (GSH) Level Measurement

A monochlorobimane (MCB) fluorescent probe (Sigma-Aldrich, St. Louis, MO, USA) was used to determine relative intracellular GSH levels as previously reported [34] with some modifications. After 24 h of treatment, the cell culture medium was removed from 3D samples and the scaffolds were transferred to 1.5 mL tubes. The cells were incubated for 15 min in DMEM with 1% FBS containing 50 μM MCB, and for a further 15 min in DMEM with 0.5 mg/mL collagenase I and 50 μM MCB (Sigma-Aldrich). Cells collected by digestion of the scaffold were centrifuged at 250× *g*. Cells were resuspended in phosphate-buffered saline (PBS) and plated on black 96-well plates. The fluorescence was measured at 355 nm (excitation) and 460 nm (emission) using a Victor Multilabel plate-reader (Perkin-Elmer, Wellesley USA). GSH levels were normalized on the base of the Crystal Violet (CV) assay. 

### 2.6. Crystal Violet Assay

Crystal Violet (CV) staining was performed as follows: Cells were fixed in 50% MeOH-PBS for 3 h at 4 °C. For 15 min at room temperature, a 0.1% (m/v) CV, 5% MeOH staining solution was incubated. The staining solution was removed and the stained cells were washed with distilled water. The plate was left to dry for 5 min under a chemical hood. The bound dye was eluted with MeOH 100% for 30 min at 4 °C. The optical density of each well was measured at 570 nm using a Victor Multilabel plate-reader (Perkin-Elmer, Wellesley USA). 

### 2.7. RNA Extraction and Real-Time PCR

Prior to RNA extraction, cell retrieval was performed by digesting the collagen scaffold in collagenase solution. Collagenase type I (Sigma-Aldrich) was dissolved in DMEM without FBS at a concentration of 0.5 mg/mL. Samples were incubated in collagenase solution for 10 min at 37 °C. Cells suspension was pelleted, and RNA was extracted with an RNeasy^®^ mini kit (Qiagen) following the manufacturer’s instruction. A total of 500 ng of RNA was used to obtain cDNA using an iScript™ cDNA Synthesis Kit (BioRad). Real-time PCR was performed using SsoAdvanced Universal SYBR Green Supermix (BioRad), and normalized expression levels were calculated relative to control cells according to the 2−ΔΔCT method. Primers were purchased from Sigma-Aldrich. The sequences are listed in Table 1. 

## 3. Results

### 3.1. Development and Characterization of the 3D SH-SY5Y Culture System 

**Figure 2:**
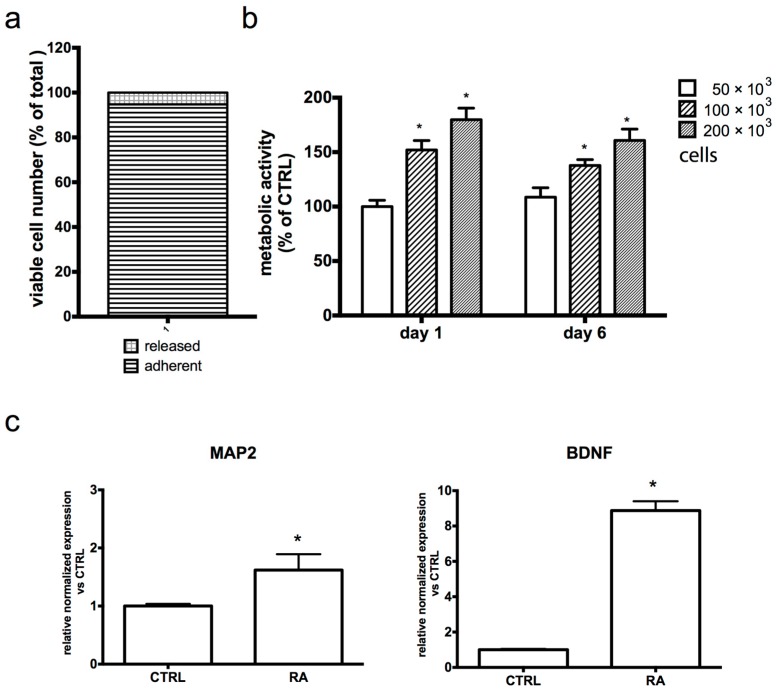
Characterization of the 3D SH-SY5Y model. (**a**) Cellular adhesion to the scaffold was evaluated 24 h after cell seeding by MTT assay as reported in Materials and Methods. Data are expressed as a percentage of total viable cells and represent the mean of three independent experiments. (**b**) Metabolic activity of the 3D model was evaluated after 1 and 6 days from cell seeding by a Prestoblue assay as reported in Materials and Methods. Each bar represents the mean ± SEM of three independent experiments. Data were analyzed with a two-way ANOVA followed by the Fisher’s test. * *p* < 0.05. (**c**) Real time-PCR was performed in the 3D culture for neuronal markers. Each bar represents the mean ± SEM of three independent experiments, which were analyzed with an unpaired T-test. * *p* < 0.05.

To fully assess biocompatibility between the collagen scaffold and SH-SY5Y cells, we evaluated cellular retention to the scaffold and cellular metabolism during 3D culture. The ability of SH-SY5Y cells to attach to the scaffold was evaluated by an MTT viability assay (Figure 2a). This assay was chosen because cells convert MTT to blue formazan that is retained by the cells and, for this reason, it makes it possible to distinguish cells attached to the scaffold from those released from the scaffold (see Figure S1). As reported in Figure 2a, about 95% of the total viable cells were able to attach after the switch from 2D to 3D culture conditions, while only 5% of the cells grew outside of the scaffold. To check the proliferation of SH-SY5Y cells, we used a Prestoblue assay as it makes it possible to monitor the metabolic activity of the same cell culture over time (Figure 2b). Cells were seeded at different concentrations, and cell viability was evaluated after 1 and 6 days. As expected, cell viability increased with an increasing numbers of cells per scaffold at both time points. Interestingly, the metabolic activity of the 3D culture after 6 days was comparable to that measured after 1 day at all tested seeding densities. Because the scaffold did not allow us to observe the cells under a microscope during growth, to verify that RA-treated SH-SY5Y cells were able to differentiate, we evaluated the mRNA level of the mature neural protein marker MAP2 as well as the secretable neurotrophin BDNF in 3D RA-treated cells (Figure 2c). Interestingly, both markers were upregulated in 3D RA-treated cells in respect to the 3D RA-untreated control, showing their ability to differentiate under 3D culture conditions. Figure S4 reports the macroscopic and microscopic appearance of the 3D SH-SY5Y culture.

### 3.2. SF, EGCG and PB Protect 3D SH-SY5Y Cells from Oxidative-Induced Injury

**Figure 3:**
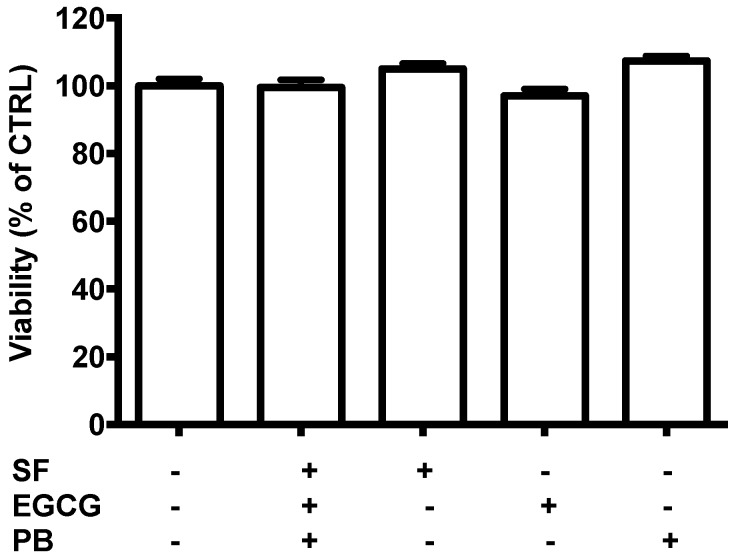
Potential cytotoxicity of sulforaphane (SF), epigallocatechin gallate (EGCG), and plumbagin (PB) on SH-SY5Y cells. Cells were treated with 1 µM SF, 2.5, µM EGCG, and 0.5 µM PB, and after 24 h, viability was evaluated by a Prestoblue assay as reported in Materials and Methods. Results are expressed as a percentage of untreated cells. Each bar represents the mean ± SEM of three independent experiments, which were analyzed with a one-way ANOVA followed by the Fisher’s test.

Before studying the neuroprotective effect of SF, EGCG, and PB, we exposed 3D differentiated SH-SY5Y cells to 1 μM SF, 2.5 μM EGCG, 0.5 μM PB, or to a combination of the three compounds at the same concentrations (SEP) (Figure 3). These concentrations were chosen according to previous reports where these concentrations were not very effective against oxidative stress [15,16,17]. Our results showed that all the tested concentrations—1 μM SF, 2.5 μM EGCG, and 0.5 μM PB—were not toxic. 

**Figure 4:**
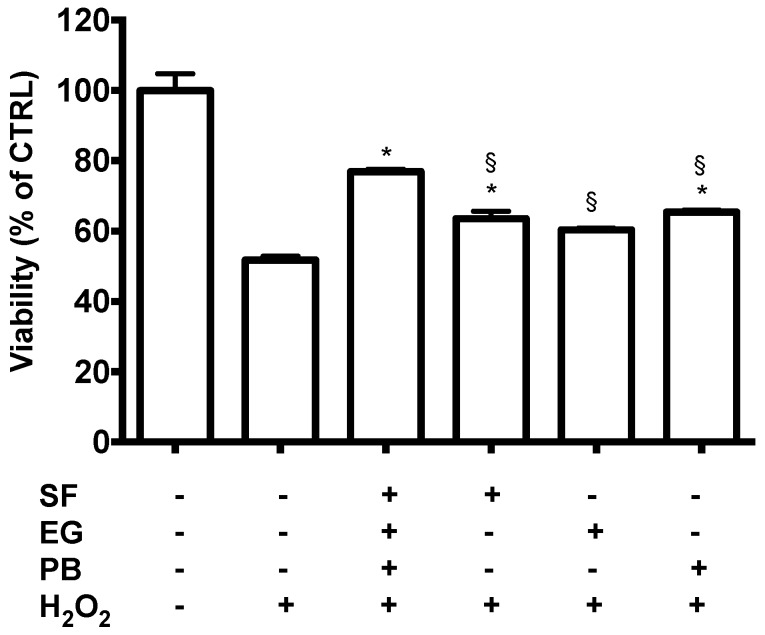
Neuroprotective activity of SF, EGCG, and PB compounds against H_2_O_2_-induced damage. Cells were treated with 1 µM SF, 2.5 µM EGCG, and 0.5 µM PB, and after 24 h, were exposed to 700 µM H_2_O_2_ to induce oxidative stress. Cell viability in 3D cultures was measured by a Prestoblue assay as reported in Materials and Methods. Data are expressed as a percentage of untreated cells. Each bar represents mean ± SEM of three independent experiments. Data were analyzed with a one-way ANOVA followed by the Fisher’s test. * *p* < 0.05 vs. H_2_O_2_ treated cells; § *p* < 0.05 vs. sulforaphane, epigallocatechin gallate, and plumbagin (SEP) co-treatment.

We then investigated the potential protective effect of the single treatments or a combination of them against H_2_O_2_-induced oxidative stress (Figure 4). As expected, incubation with 700 µM H_2_O_2_ for 2 h induced a significant reduction of cell viability compared to the control cells (Figure S2). Only 0.5 µM PB, 1 µM SF, and the co-treatment (1 µM SF, 2.5 µM EGCG, and 0.5 µM PB) were able to protect the cells against H_2_O_2_-induced damage. Of note, SEP co-treatment was the most effective treatment as it significantly increased cell viability compared to the other treatments.

### 3.3. SEP Co-Treatment Enhances Antioxidant Defenses 

**Figure 5:**
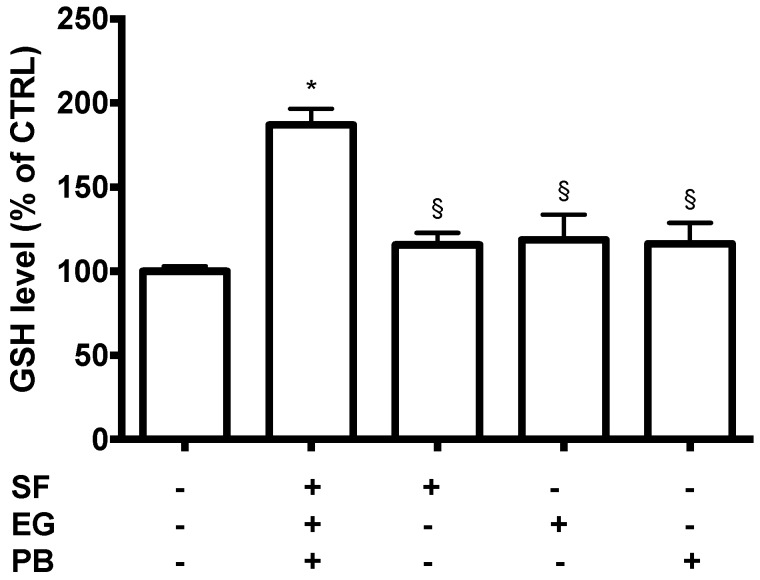
Antioxidant activity of SF, EGCG, and PB compounds on SH-SY5Y cells. Cells were treated with 1 µM SF, 2.5 µM EGCG, and 0.5 µM PB, and after 24 h GSH levels were evaluated with an monochlorobimane (MCB) assay as reported in Materials and Methods. Data are expressed as a percentage of untreated cells (CTRL). Each bar represents mean ± SEM of three independent experiments. Data were analyzed with a one-way ANOVA followed by the Fisher’s test. * *p* < 0.05 vs. untreated cells; § *p* < 0.05 vs. SEP co-treatment.

As our results showed a higher neuroprotective activity of SEP co-treatment (1 µM SF, 2.5 µM EGCG, and 0.5 µM PB) compared to the single treatments of 1 µM SF, 2.5, µM EGCG, or 0.5 µM PB, we investigated the ability of SEP co-treatment to modulate the cellular redox state by evaluating GSH levels with an MCB assay. The effect of the different treatments after 24 h on GSH levels is reported in Figure 5. All the treatments were able to significantly increase GSH levels in respect to control cells. In agreement with the viability data, we observed the most effective increase of GSH levels after SEP co-treatment (1 µM SF, 2.5 µM EGCG, and 0.5 µM PB) in comparison to the single treatment of 1 µM SF, 2.5 µM EGCG, or 0.5 µM PB. 

### 3.4. SEP Co-Treatment Modulates Genes Involved in Oxidative Stress Control 

**Figure 6:**
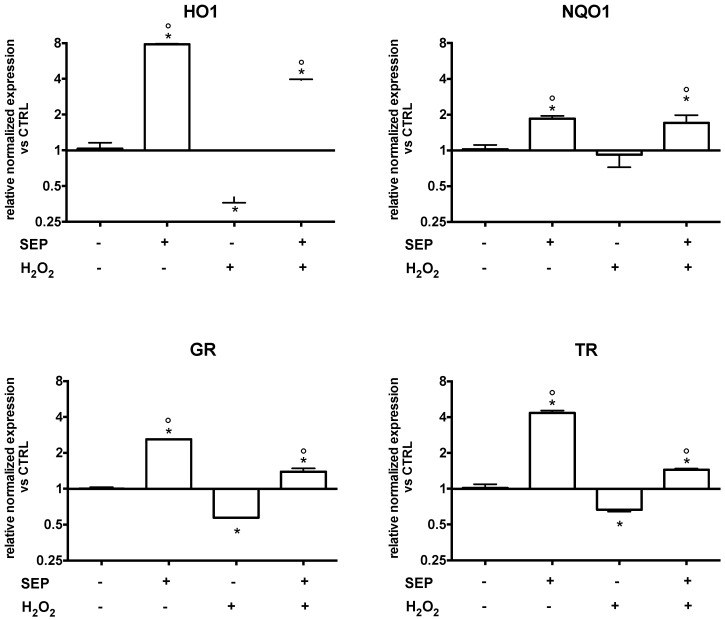
Effect of SEP co-treatment on antioxidant enzyme expression. Cells were co-treated with 1 µM SF, 2.5 µM EGCG, and 0.5 µM PB for 6 h. Oxidative stress was induced with 700 µM H_2_O_2_ for 1 h prior to lysis. Real time-PCR was performed to detect heme oxygenase 1 (HO1), NADPH: quinone oxidoreductase 1 (NQO1), glutathione reductase (GR), and thioredoxin reductase (TR) mRNA levels. Data are expressed as relative abundance compared to untreated cells. Each bar represents mean ± SEM of three independent experiments. Data were analyzed with a one-way ANOVA followed by the Fisher’s test. * *p* < 0.05 vs. untreated cells, ° *p* < 0.05 vs. H_2_O_2._

As the previous data showed that SEP co-treatment was significantly more effective compared to the single treatments, we decided to study its ability to modulate cellular antioxidant status. Real-time PCR analysis was employed to investigate the ability of SEP co-treatment to modulate the mRNA level of different antioxidant enzymes. The cDNA was obtained from 3D SH-SY5Y cultures that were co-treated (1 µM SF, 2.5 µM EGCG, and 0.5 µM PB) for 6 h. The 3D cultures were then exposed to 700 µM H_2_O_2_ for 1 h prior to lysis (Figure 6). Importantly, SEP co-treatment induced a significant and marked upregulation of heme oxygenase 1 (HO1), NADPH: quinone oxidoreductase 1 (NQO1), glutathione reductase (GR), and thioredoxin reductase (TR) in 3D cultures although with different levels of upregulation (Figure 6). Moreover, SEP co-treatment in the presence of oxidative stress induced a significant upregulation of all tested genes in respect to H_2_O_2_-treated cells. 

**Figure 7:**
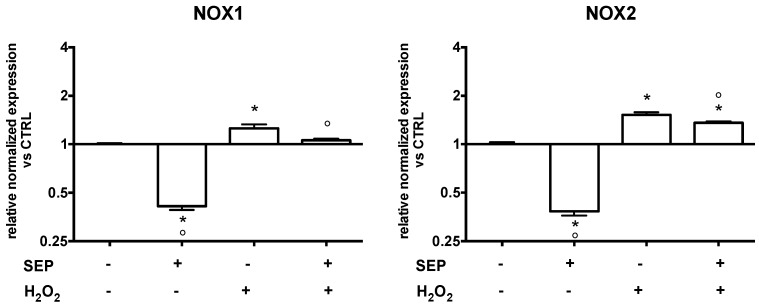
Effect of SEP co-treatment on NADPH oxidase 1 (NOX1) and NADPH oxidase 2 (NOX2). Cells were co-treated with 1 μM SF, 2.5 μM EGCG, and 0.5 μM PB for 6 h. Oxidative stress was induced with 700 µM H_2_O_2_ for 1 h prior to lysis. Real time-PCR was performed to detect NOX1 and NOX2 mRNA levels. Data are expressed as relative abundance compared to untreated cells. Each bar represents mean ± SEM of three independent experiments. Data were analyzed using a one-way ANOVA followed by the Fisher’s test. * *p* < 0.05 vs. untreated cells, ° *p* < 0.05 vs. H_2_O_2._

NADPH oxidase (NOX) enzymes have been shown to be a major source of ROS in the brain and to be involved in several neurological diseases [35]. On this basis, we studied the modulatory effect of SEP co-treatment on NOX1 and NOX2 expression using real-time PCR analysis (Figure 7). In the absence of oxidative stress, SEP co-treatment had a strong effect on these enzymes as it significantly reduced NOX1 and NOX2 expression compared to untreated cells. In the presence of oxidative stress (700 µM H_2_O_2_), SEP co-treatment significantly reduced NOX1 and NOX2 expression compared to H_2_O_2_-treated cells. Of note, SEP co-treatment before peroxide exposure maintained NOX1 levels at a value comparable to control cells. 

### 3.5. SEP Co-Treatment is able to Modulate Insulin-Degrading Enzyme (IDE) Gene Expression 

**Figure 8:**
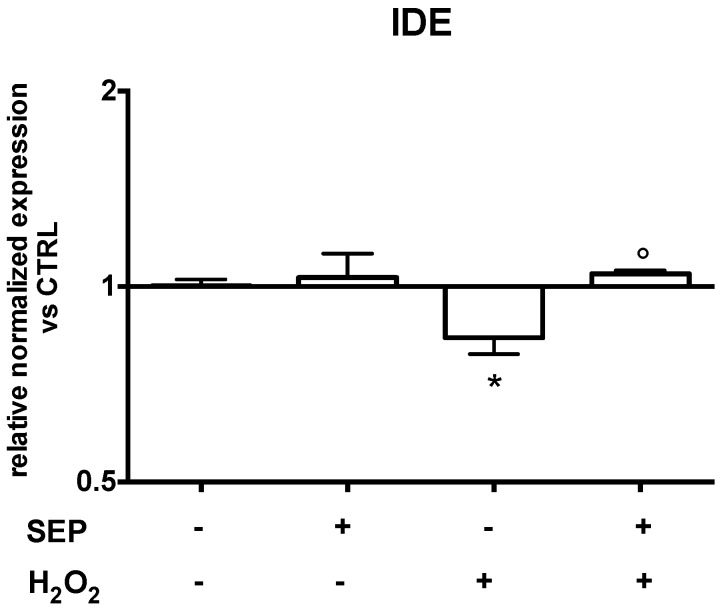
Effect of SEP co-treatment on insulin-degrading enzyme (IDE). Cells were co-treated with 1 μM SF, 2.5 μM EGCG, and 0.5 μM PB for 6 h. Oxidative stress was induced with 700 µM H_2_O_2_ for 1 h prior to lysis. Real time-PCR was performed to detect IDE mRNA levels. Data are expressed as relative abundance compared to untreated cells. Each bar represents mean ± SEM of three independent experiments. Data were analyzed with a one-way ANOVA followed by the Fisher’s test. * *p* < 0.05 vs. untreated cells, ° *p* < 0.05 vs. H_2_O_2_.

To investigate if SEP co-treatment had other neuroprotective activities besides the antioxidant one, we studied its effect on insulin-degrading enzyme (IDE) expression. IDE plays a significant role in Aβ degradation [36], which is one of the main hallmarks of Alzheimer’s disease. Moreover, recent studies have demonstrated that increasing Aβ degradation as opposed to inhibiting synthesis is a more effective strategy for preventing Aβ build-up [37]. In our 3D SH-SY5Y cultures, IDE mRNA levels were downregulated by oxidative stress, but, interestingly, SEP co-treatment (1 μM SF, 2.5 μM EGCG, and 0.5 μM PB) was able to upregulate its expression at levels comparable to untreated cells (Figure 8). 

## 4. Discussion 

The prevalence of neurodegenerative disorders is growing [2,38] in parallel to the urgency to find new compounds for the treatment of such diseases, in which oxidative stress is a common hallmark and has been suggested to play a causative role [39,40]. Unfortunately, the screening of drug leads and natural compounds to counteract neurodegeneration using 2D cell cultures often results in the unsuccessful translation of data to clinics. Neurons are strongly influenced by their immediate extracellular environment, and there is a great need to develop new culture systems that more faithfully reproduce the complexity of this milieu in vivo. Human 3D cell culture models are a good alternative to animal models [41,42]. In contrast to 2D cell cultures, 3D cell cultures do not overlook the physical interactions existing between cell–cell and cell–matrix and have a higher resemblance to the in vivo phenotype. Ideal scaffolds for neuronal tissue or disease modelling should exhibit suitable 3D architecture for in vitro manipulation, should facilitate cell adhesion while promoting neurites outgrowth, and have high biocompatibility [43]. Collagen type I is highly used as scaffold because of its abundance and ubiquity in most of the hard and soft tissues in the human body [44]. Porous collagen sponges have been used to grow various cell types in vitro [45] and collagen derived scaffolds have been widely used in neural tissue engineering for drug delivery [46]. Furthermore, the extracellular matrix (ECM) in nerves is mainly composed of type I collagen [47] and is a commonly used material in nerve tissue engineering [47] and for peripheral nerve regeneration [48]. ECM geometry and composition are well known to influence cell morphology and gene expression. It has been shown that SH-SY5Y cells extended longer neurites in 3D collagen I hydrogel cultures than in 2D cultures [26]. On this basis, we used equine native collagen, commercially available for clinical application, as scaffold to support 3D cultures of differentiated SH-SY5Y cells. 

Our aim was to study the neuroprotective activity of a combination of SF, EGCG, and PB in counteracting peroxide-induced damage in 3D cultures of differentiated SH-SY5Y cells.

Taking into account previous studies showing the protective effects of these compounds against oxidative stress [49,50], we decided to treat SH-SY5Y cells with specific concentrations of SF, EGCG, and PB to better mimic concentrations that could be measured in plasma after oral intake of the three compounds [51]. We selected a porous instead of a hydrogel scaffold because it easily permits the removal of apoptotic blebs and dead cells by washing during medium exchange. The used collagenous scaffold was found to be highly biocompatible since it supported the adhesion and proliferation of SH-SY5Y cells in the 3D environment. 

Our data demonstrated that SEP co-treatment was significantly more effective against oxidative stress than the single treatments of PB, EGCG, or SF, suggesting a synergistic protective mechanism of the co-treatment. In particular, SEP was more effective in limiting cell injury induced by H_2_O_2_ exposure. These data were also demonstrated in our 2D cell model, and we confirmed the superior efficacy in enhancing GSH levels by SEP co-treatment compared the single treatments both in 2D and 3D models (Figure S3). Although different reports have discussed the neuroprotective effect of PB, EGCG, and SF against brain-induced toxicity [52,53,54], there is no documented work on the effect of their combination. Our results are in agreement with other papers demonstrating the synergistic protective effect of different combinations of natural compounds against neurodegeneration [55,56,57]. In general, the superior protection of co-treatments compared to the single treatments could be probably ascribed to the concurrent modulation of different molecular targets involved in the pathogenesis and progression of these multi-factorial diseases. 

To better elucidate the mechanisms behind SEP protection against H_2_O_2_ in SH-SY5Y cells, we investigated the effect of the co-treatment on the expression of different antioxidant enzymes: heme oxygenase 1 (HO1), NADPH: quinone oxidoreductase 1 (NQO1), glutathione reductase (GR) and thioredoxin reductase (TR). 

The enzyme HO1 converts heme to three end products, namely biliverdin, CO, and ferrous ion [58], then biliverdin reductase activity produces the antioxidant bilirubin. NQO1 is a highly inducible detoxifying flavoenzyme. It catalyzes the reduction of quinones generating stable hydroquinones and possesses superoxide scavenging activity [59]. GR is responsible for maintaining a storage amount of reduced glutathione [60]. The thioredoxin (Trx) system, composed of Trx, TR, and NADPH as a cofactor, is a cellular defense system that is ubiquitously involved in converting ROS to nontoxic metabolites [61]. In such a system, the Trx in reduced status can be oxidized into oxidized Trx during the degradation of H_2_O_2_ and then reduced by TR [62]. 

SF is known to upregulate antioxidant defense through the induction of HO1, NQO1, and GR in SH-SY5Y cells [15,63], while GR, TR, and NQO1 have been observed to be upregulated in cortical neurons [64]. EGCG induced HO1 expression in rat-cultured neurons [65] and increased protein levels in treated rats following focal cerebral ischemia [66]. PB treatment led to increased levels of HO1, NQO1, and TR in SH-SY5Y cells [17]. Interestingly, our data showed that SEP co-treatment, in the absence of oxidative stress, strongly upregulated these enzymes compared to control cells (the same results were also obtained in the 2D model (Figure S3)), and, in the presence of oxidative stress, it was able to significantly increase the expression of these enzymes compared to H_2_O_2_ exposed cells. In agreement with our data that showed the enhanced effect of a combination of different compounds compared to treatment with the single compounds, in a previous work, we observed that a combination of EGCG and SF counteracts in vitro oxidative stress and delays stemness loss of amniotic fluid stem cells [67]. Moreover, a combination of berberine with resveratrol had enhanced hypolipidemic effects in high fat diet-induced mice and was able to decrease the lipid accumulation in adipocytes to a level significantly lower than that of the treatment with the single compounds [68]. 

Recently, it has been suggested that inhibition of the generation, rather than the scavenging, of ROS may be a more successful strategy to counteract oxidative stress-induced neurodegeneration [69]. ROS can be produced by many enzymes in the cells such as mitochondria respiratory complexes, NADPH oxidase, nitric oxide synthase, cytochrome 450, cyclooxygenase, lipoxygenase, and xanthine oxidase [14,70]. Interestingly, all these enzymes except NADPH oxidase produce ROS as a byproduct, while NOX enzymes generate ROS as a principal aim [71,72,73]. Moreover, different studies have shown the involvement of NADPH oxidase family members in brain injury and neurodegenerative disorders (reviewed in [69]). Our data demonstrated that SEP co-treatment was effective in reducing NOX1 and NOX2 expression compared to control cells and was also able to counteract the increase of NOX1 and NOX2 expression induced by H_2_O_2_. This means that SEP not only potentiates the antioxidant defense system upregulating fundamental enzymes, but also reduces the intracellular production of ROS. 

The last aim of this paper was to investigate if SEP could modulate other hallmarks of neurodegeneration in addition to its ability to counteract oxidative stress. We decided to focus our attention on IDE, the main protease responsible for amyloid β clearance [74,75,76]. A reduction of IDE activity in the brain with age and during the early stages of Alzheimer’s disease (AD) has been observed [74], suggesting that IDE downregulation may be among the triggers of AD. Of note, SEP counteracted the strong downregulation of IDE induced by oxidative stress, maintaining IDE expression at a level comparable to control cells, suggesting a potential role of SEP in counteracting AD.

In conclusion, we highlighted that an appropriate synergistic combination of natural antioxidants such as SF, EGCG, and PB can help to rescue neuronal cells from oxidative stress cell death. The protective effect of the co-treatment was observed in a novel 3D model of SH-SY5Y cells that we developed. In agreement with other authors [77,78,79], we suggest that a 3D culture system better mimics cell–cell interactions and cell–ECM interactions compared to the traditional 2D monolayer. In particular, our 3D model would be useful for future investigations of the neuroprotective activity of natural compounds [80]. In the present study, we observed the protective effect of an “acute” co-treatment with SF, EGCG, and PB but, taking into account the nature of neurodegeneration, a subchronic/chronic administration should be even more effective. For this reason, future studies will have to be carried out to investigate the effect of chronic SEP treatment against oxidative stress in neurodegeneration. The present findings underscore the importance of a combinatorial approach for effective treatments against oxidative damage in neurodegeneration. Moreover, 3D SH-SY5Y cell culture systems appear to be the ideal environment for in vitro assays regarding the effects of phytochemicals on cell viability.

## Figures and Tables

**Table 1:** Primer sequences.

Gene	Sequence	RefSeq Accession n.
RPS18 *	Fw CAGAAGGATGTAAAGGATGG	NM_022551
	Rv TATTTCTTCTTGGACACACC	
MAP2	Fw GAAGATTTACTTACAGCCTCG	NM_002374
	Rv GGTAAGTTTTAGTTGTCTCTGG	
BDNF	Fw CAAAAGTGGAGAACATTTGC	NM_001143811
	Rv AACTCCAGTCAATAGGTCAG	
HMOX1(HO1)	Fw CAACAAAGTGCAAGATTCTG	NM_002133.2
	Rv TGCATTCACATGGCATAAAG	
IDE	Fw CAACCTGAAGTGATTCAGAAC	NM_001165946
	Rv AATATGTGGTTTCACAAGGG	
NOX1	Fw CCGGTCATTCTTTATATCTGTG	NM_007052
	Rv CAACCTTGGTAATCACAACC	
NOX2	Fw AAGATCTACTTCTACTGGCTG	NM_000397
	Rv AGATGTTGTAGCTGAGGAAG	
NQO1	Fw AGTATCCACAATAGCTGACG	NM_000903
	Rv TTTGTGGGTCTGTAGAAATG	
GSR (GR)	Fw GACCTATTCAACGAGCTTTAC	NM_000637
	Rv CAACCACCTTTTCTTCCTTG	
TXNRD1 (TR)	Fw AGACAGTTAAGCATGATTGG	NM_001093771
	Rv AATTGCCCATAAGCATTCTC	

* reference gene.

## Supplementary Materials

The following are available online at https://www.mdpi.com/2076-3921/8/10/420/s1. Figure S1: Visual explanation of MTT assay as used in this study, Figure S2: Cell viability of SH-SY5Y incubated with H_2_O_2_, Figure S3: Results obtained with SEP co-treatement in SH-SY5Y 2D model. Figure S4: Images of SH-SY5Y 3D model.

## Abbreviations

2D	Two-dimensional
3D	Three-dimensional
BDNF	Brain-derived neurotrophic factor
DCFH-DA	2,7-dichlorodihydrofluorescein diacetate
DMEM	Dulbecco’s Modified Eagle’s medium
DMSO	Dimethyl sulfoxide
ECM	Extra cellular matrix
EGCG	Epigallocatechin gallate
GR	Glutathione reductase
GSH	Reduced glutathione
H_2_O_2_	Hydrogen peroxide
HO1	Heme oxygenase 1
IDE	Insulin-degrading enzyme
MAP2	Microtubule-associated protein 2
MCB	Monochlorobimane
MTT	3-(4,5-dimethylthiazol-2-yl)-2,5-diphenyl-tetrazolium bromide)
NOX1	NADPH oxidase 1
NOX2	NADPH oxidase 2
NQO1	NAD(P)H: quinone oxidoreductase 1
PB	Plumbagin
PBS	Phosphate buffered saline
PCR	Polymerase chain reaction
RA	All-trans retinoic acid
ROS	Reactive oxygen species
RPS18	Ribosomal protein S18
SEP	Sulforaphane, Epigallocatechin gallate, Plumbagin
SF	Sulforaphane
TR	Thioredoxin reductase 1
