# Supplementary material for: Combined Treatment with Three Natural Antioxidants Enhances Neuroprotection in a SH-SY5Y 3D Culture Model
Source: Antioxidants (Basel). 2019 Sep 20;8(10):420. doi: 10.3390/antiox8100420 (PMC6827135; doi:10.3390/antiox8100420)
Supplement: Supplementary file 1 [file antioxidants-08-00420-s001.pdf]

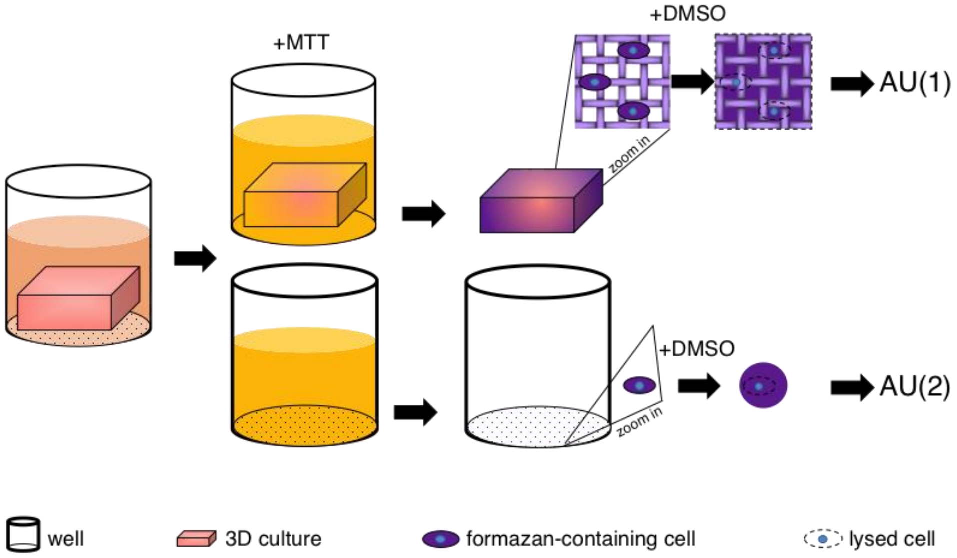

Figure 1. Prior to adding MTT, 3D cultures were transferred to clean cell culture wells. MTT 0.5 mg/mL was prepared in cell medium and added to the 3D cultures. To measure cells that did not attach to the scaffold, MTT was also added to the wells used during the seeding. MTT solution was incubated for 2 h at 37°C, 5% CO_2. After removing MTT solution, DMSO was added to samples to lyse the cells, for 20 min with agitation (200 rpm). The absorbance of formazan was measured at 595 nm using a microplate spectrophotometer VICTOR3 V Multilabel Counter (Perkin-Elmer). 100% of the signal is considered the sum of the two respective absorbance values, the first deriving from the primary wells used during the seeding and the second deriving from scaffolds.

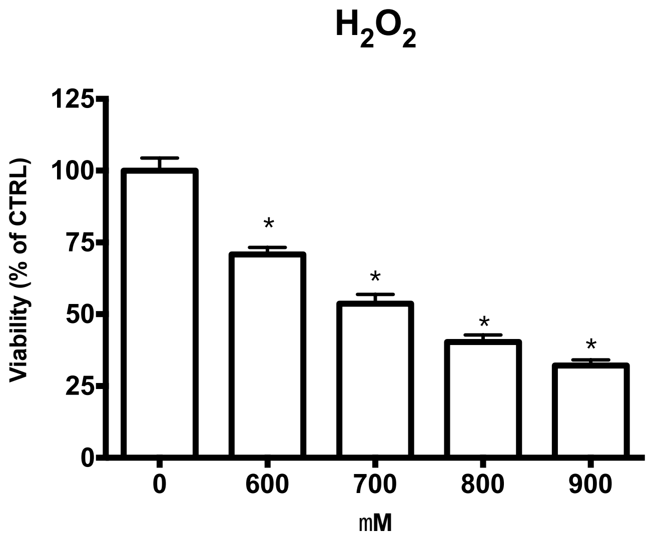

Figure 2. 3D culture were treated with peroxide (0.6–0-9 μM) for 2 hours. Cell viability was measured by Prestoblue assay. 700 μM has been chosen to induce oxidative stress as it reduces cell viability by 50% with respect to control cells (0 μM). Similar H_2O_2 concentration has been recently used by Giusti et al. 2018 [33] in 2D differentiated SH-SY5Y cells.

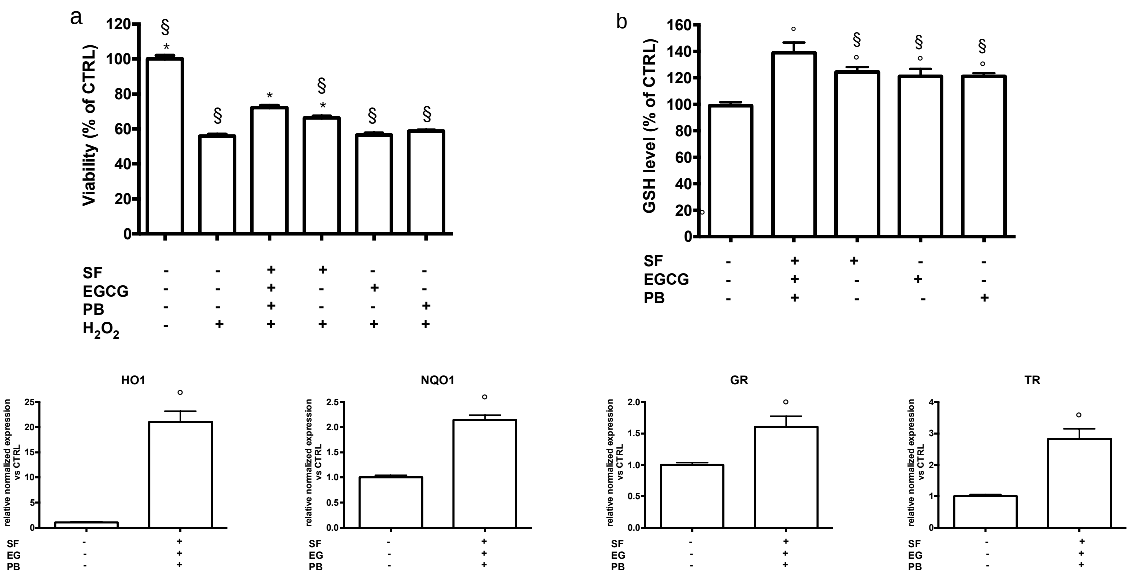

Figure 3. Neuroprotective and antioxidant activity of SEP against H_2O_2-induced damage in 2D culture. (a) Cells were treated with 1 μM SF, 2.5 μM EGCG and 0.5 μM PB and after 24 h were exposed to H_2O_2 to induce oxidative stress. Cell viability was evaluated by Prestoblue assay. Data are expressed as percentage of untreated cells. Each bar represents mean ± SEM of three independent experiments. Data were analysed by one-way ANOVA followed by Fisher's test. *p< 0.05 vs H_2O_2 -treated cells. §p<0.05 vs co-treated cells. (b) Cells were treated with 1 μM SF, 2.5 μM EGCG and 0.5 μM PB and for 24 h. GSH levels were evaluated by MCB assay as reported in Material and Methods section. Data are expressed as percentage of untreated cells. Each bar represents mean ± SEM of three independent experiments. Data were analysed by one-way ANOVA followed by Fisher's test. °p< 0.05 vs untreated cells. §p<0.05 vs co-treated cells. (c) Cells were co-treated with 1 μM SF, 2.5 μM EGCG and 0.5 μM PB for 6 h. Realtime-PCR was performed to detect HO1, NQO1, GR and TR mRNA levels. Data are expressed as relative abundance in respect to controls. Each bar represents mean ± SEM of three independent experiments. Data were analysed by unpaired T-test. °p<0.05 vs untreated cells.

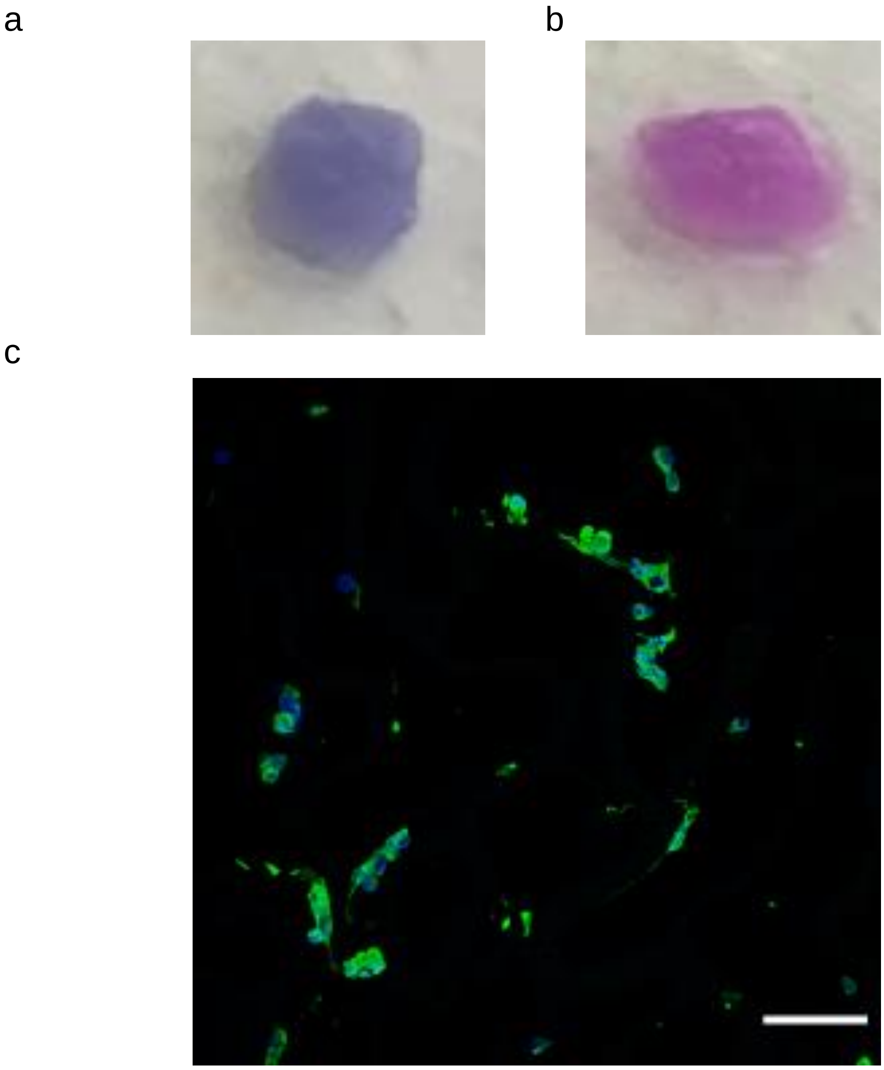

Figure 4. Macroscopic and microscopic appearance of 3D SH-SY5Y culture. (a) Digital image of a cell-free scaffold after Prestoblue viability assay. (b) Digital image of a scaffold seeded with cells after Prestoblue viability assay. (c) Fluorescence microscopy on SH-SY5Y 3D culture histological section. Green fluorescence is anti-β-tubulin III staining for neuronal cells. Blue fluorescence is DAPI staining for nuclei. Scale bar is 50 μm.
